# Methods and Applications of 3D Ground Crop Analysis Using LiDAR Technology: A Survey

**DOI:** 10.3390/s23167212

**Published:** 2023-08-16

**Authors:** Matias J. Micheletto, Carlos I. Chesñevar, Rodrigo Santos

**Affiliations:** 1 Golfo San Jorge Research and Transfer Center (CIT-GSJ), CONICET, Comodoro Rivadavia 9000, Argentina; matias.micheletto@uns.edu.ar; 2 Department of Computer Science and Engineering, UNS, ICIC-CONICET-UNS, Bahia Blanca 8000, Argentina; cic@cs.uns.edu.ar; 3 Department of Electrical Engineering and Computers, UNS, ICIC-CONICET-UNS, Bahia Blanca 8000, Argentina

**Keywords:** agriculture, smart farming, Light Detection and Ranging (LiDAR), ground crops

## Abstract

Light Detection and Ranging (LiDAR) technology is positioning itself as one of the most effective non-destructive methods to collect accurate information on ground crop fields, as the analysis of the three-dimensional models that can be generated with it allows for quickly measuring several key parameters (such as yield estimations, aboveground biomass, vegetation indexes estimation, perform plant phenotyping, and automatic control of agriculture robots or machinery, among others). In this survey, we systematically analyze 53 research papers published between 2005 and 2022 that involve significant use of the LiDAR technology applied to the three-dimensional analysis of ground crops. Different dimensions are identified for classifying the surveyed papers (including application areas, crop species under study, LiDAR scanner technologies, mounting platform technologies, and the use of additional instrumentation and software tools). From our survey, we draw relevant conclusions about the use of LiDAR technologies, such as identifying a hierarchy of different scanning platforms and their frequency of use as well as establishing the trade-off between the economic costs of deploying LiDAR and the agronomically relevant information that effectively can be acquired. We also conclude that none of the approaches under analysis tackles the problem associated with working with multiple species with the same setup and configuration, which shows the need for instrument calibration and algorithmic fine tuning for an effective application of this technology.

## 1. Introduction

LiDAR (an acronym standing for “Light Detection and Ranging”) is a popular remote-sensing method used for measuring the exact distance of an object’s surface. LiDAR’s first applications in the 1960s were in meteorology when laser scanners were mounted in aircraft being used by the National Center for Atmospheric Research to measure clouds and pollution [1].

Nevertheless, LiDAR did not deserve much attention until many years later with the introduction of the Global Positioning System (GPS) [2].Since then, LiDAR has become a popular method for calculating accurate geospatial measurements. Nowadays, its scope has spread across a wide range of research areas, including autonomous vehicles [3], coast management systems [4], wind speed prediction [5], archeological surveying [6], and particularly in agriculture, among many others.

This ranging technology is based on the use of a pulsed laser beam to calculate a target’s distance from the sensor’s detector [2]. These light pulses—put together with the information collected by the reference system—generate accurate 3D information about the target’s surfaces and visible shape; this is the main characteristic of LiDAR.

For agriculture activities, LiDAR systems cover a wide spectrum of applications [7,8]. Generally speaking, LiDAR scanning of crop fields allows for acquiring an accurate and quantifiable model of plant structures, which makes it possible to perform any type of analysis indirectly and without resorting to methods that are traditionally destructive. This allows, for example, to quickly measure the height and estimate the density of a crop in an extensive way, estimate harvests and yields, and control robots or agricultural machinery being partially or completely autonomous, paving the way to even more complex applications (such as pests or diseases detection or analysis of the need for irrigation and fertilization).

Apart from the mentioned applications, the morphological analysis of ground crops involves many aspects of agronomic interest. The volume and shape of plants can be seen as a reflection of the state of the soil, which is the main resource in the context of agricultural production. In regions where soils are exposed to heavy erosive processes, preserving an adequate vegetation cover is a possible approach to preserving or improving the soil quality [9].

The use of LiDAR technology in agriculture has been reviewed before; for instance, the work presented in [10] focuses on the main findings and pending challenges for each one of the items of the analyzed bibliography, and the main difference with our contribution lies in the fact that the classification proposed by the authors has different criteria, as it only considers UAV-mounted sensors and does not take into account ALS mounted on manned aircraft, which was a widely used method until the popularization of smaller and accessible UAV units. The review presented in [10] is broader in the sense that it also takes into account shrubs and other tree species, such as apple and olive plants, sugar cane, or vineyards, and only the most relevant research articles are included in the literature review.

In this work, we attempt to summarize what the scientific community has performed in 2005 to 2022 in terms of three-dimensional ground crop scanning using LiDAR technology and to provide an overview of the necessary tools to carry out new contributions in this emerging field. The article is structured as follows: In Section 2, the methodology employed during the systematic review is explained. Section 3 details the emerging taxonomic categories resulting from our review. In Section 4, the main difficulties and challenges that the original authors encountered during their research are listed along with some alternatives to overcome them. Finally, Section 5 presents the final conclusions obtained, also discussing some possible future research lines.

## 2. Methodology

We performed a comprehensive survey of the existing bibliography on LiDAR technologies starting with two major scientific databases for computer science research (namely IEEE Xploreand ScienceDirect). Using the keywords “LiDAR” and “Agriculture”, we identified an initial list of 15 papers, which was expanded by computing the transitive closure of the different references associated with those papers. In other words, bibliographical entries referring to LiDAR associated with papers from the initial list were analyzed, as well as the references associated with those entries.

Additionally, it must be pointed out that research papers involving trees and shrubs were not taken into account, focusing only on those in which the associated crop corresponded to herbs and/or vegetation at ground level. As a result, we ended up with a total of 53 papers, and research articles which are listed in Table A1 of the appendix, at the end of this article.

In order to analyze the collection of papers under study, the results were sorted according to the categories listed in Table 1. Each of the taxonomic aspects that were taken into account from the reviewed bibliography is detailed in the following section.

## 3. Classifying LiDAR Technology: A Taxonomic View

From the analysis of the reviewed articles, we could identify the different categories mentioned in the previous section (application areas, crop species, scanner technology, mounting platform, additional sensors and instrumentation, and LiDAR software). Such categories provide a taxonomic view of LiDAR technology, helping to better understand the key points that must be taken into account when carrying out a new study that involves the use of LiDAR scanners in ground crops. The different criteria were selected considering the impact they have on the development of this type of scanner. When considering a range of possible application areas, it is clear that the main motivation to innovate in these sensors is related to the different application possibilities they have. As in the previous case, considering the existence of different crop species, as they differ in size and shape, it is fundamental to consider them for the development of the scanner. The third axis refers to the technology used and here it is important as not all wavelengths have similar performances and should be selected in accordance with the application to which the scanner is oriented. The mounting platform is also an important aspect, as it must consider the associated technology and application area. The possibility of combining several sensors in the equipment provides additional value to the scanner and is evaluated in that direction, whereas the last axis, the software, is a key issue as it is the element that processes the data, transforming it into information. To ease the understanding of some terms mentioned by the original authors, Table 2 provides a complete list of acronyms that were found in the reviewed bibliography. In what follows, we discuss in detail all of the previous categories.

### 3.1. Application Areas

By application, we refer to the potential problems that LiDAR technology solves when applied to the 3D scanning and reconstruction of ground crops. Some of the reviewed articles focus on the main aspects and challenges of reconstructing the three-dimensional model of the crops with the highest accuracy possible, leaving the results open to many potential applications. We categorize these papers as “general purpose 3D reconstruction”. In other cases, it is intended to extract a set of specific parameters from the acquired model having agronomic interest (e.g., the height of crops or the Leaf Area Index (LAI)), where a high-resolution model is not mandatory, but rather an appropriate data-processing technique. Finally, we identify a third group of applications where the goal is to achieve automatic control of certain processes, such as automatic irrigation or fertilization systems. This is the case of [11,12,13,14].

As an outcome of the above analysis, we established three categories to characterize the reviewed articles according to their application area:

- **General purpose** 3D reconstruction.
- **Parameter characterization** of crops.
- Agriculture machinery **automatic control**.

The bold highlighted text indicates the keyword used to identify the application, to be used later in Table A1.

### 3.2. Analyzed Crop Species

**Figure 1:**
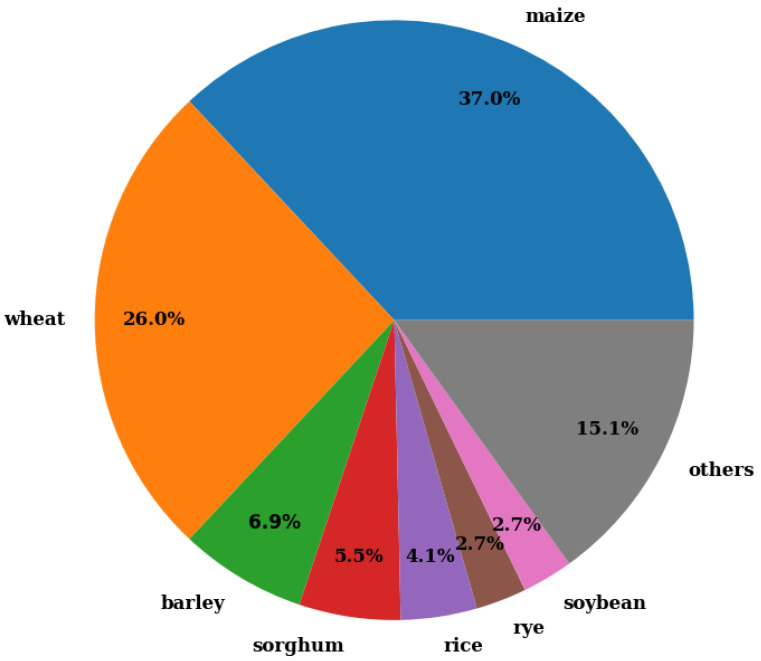
Species involved in the reviewed bibliography.

The species of ground crops involved in the different studies and the frequency of occurrence in the analyzed bibliography are slightly correlated with the statistics of world crop production, being maize in the first place, followed by other cereals such as wheat, rye, or barley, also including sorghum, rice, and soybean [15]. The list of ground crops also includes sunflower [16], oat [17], sugar beet [18], Miscanthus giganteus [19], fescue [20], cotton [21], American mint [22], and peas [23]. Figure 1 shows the proportion of each crop that was used as species under study. At this point, it is important to emphasize again that the choice of species of different crops that were analyzed using LiDAR technology was made based on requirements that arise from the productive sector, so the fact that the crops that were most studied are those that occupy the largest proportion of cultivated land at a global level.

### 3.3. LiDAR Scanner Technology

There are two main types of LiDAR scanning technologies: those in which the working principle is based on *time of flight* (ToF) and the ones based on *phase shift* (PS). The first type usually has a larger measurement range (up to 6 km), whereas the latter has a higher accuracy and speed (up to 2 million points per second) [24]. For ToF-based scanners, there are two categories: *discrete return* and *full waveform*. Discrete return is based on proprietary algorithms used to extract the range and energy of one or more targets along the laser beam’s path. On the other hand, full waveform systems record all the reflected energy as a function of range, giving a more complete description of the scattering event and allowing a more accurate measurement of target properties over diffuse targets such as vegetation [25].

Regarding the scanning devices, we have taken into consideration different aspects that may be of interest to a researcher or practitioner when deciding what instrument to use. The top applied devices are SICK LMS400 and Leica ALS70 (with five articles each). The first one is a phase shift terrestrial laser scanner (TLS) with a maximum range of 3 m, whereas the latter is an airborne scanner with a nominal range (flight level) of 3500 m.

Table 3 shows the summarized specifications of the top ten most used devices mentioned in the bibliography. The specifications were extracted from online datasheets provided by manufacturers and also from other scientific publications [26]. Note that the row corresponding to the Pulstec TDS-130L scanner, used in [27,28,29], is incomplete (since the online datasheet for this device was not available for access at the time this article was written).

Most parameters listed in the table are configurable and may vary depending on scanning conditions. For example, the range and accuracy are affected by the target reflectance, an issue that will be detailed in Section 4.1. For the case of power consumption, the nominal values were selected for normal operating conditions, since some instruments have different power modes or heating plates to operate in very cold environments. In a similar way, the weight of each instrument considers the main device only without accessories that can be attached to the scanning platform (such as lenses, tripods, heating plates, stabilizers, and many others).

### 3.4. Mounting Platform

There exist many ways to mount the LiDAR instrument in order to perform the scanning of ground crops. At this point, we propose a taxonomic classification scheme to organize the different categories. The most relevant categories in LiDAR devices are *terrestrial* and *airborne*. The first one usually intends to cover small areas whereas the latter allows for the scanning of larger surfaces (but usually with smaller accuracy). An exhaustive comparison of both techniques when applied to land surface scanning was made in [30].

From the analyzed articles, there were eight papers that used aircraft as an airborne transportation method, whereas the remaining three used UAVs. On the other hand, for the case of terrestrial mount, depending on the change in the position of the laser scanner during the scanning process, we classify the scanning platforms as *stationary* or *mobile*. Both methods were used in almost the same proportion in the research work under analysis. If the laser scanner moves or rotates within a small stationary platform, we classify it as stationary. In order to perform the motion during the mobile scanning, a mobile mount is required which can be a custom structure or platform, an autonomous rover, an ATV, a passenger car, or agriculture machinery.

**Figure 2:**
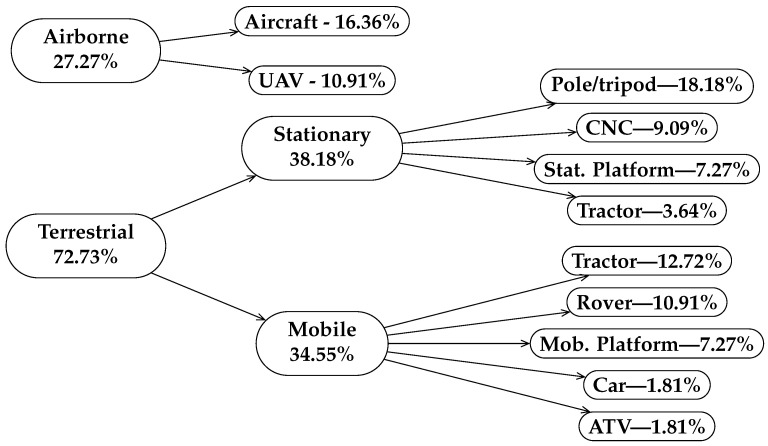
Hierarchy of scanning platforms and percentages of articles in which they were used.

Figure 2 shows the hierarchical classification of the different scanning platforms and the proportion of papers in which each method was used. Note that for some cases the sum of percentages of child classes does not match the proportion associated with the parent class (since some of the papers combine more than one platform type, whereas others do not clarify the way the scanner was mounted).

Aircraft-mounted LiDAR systems provide wide coverage and high-altitude data collection capabilities, making them suitable for large-scale surveying and mapping of plots or parcels of land. They can cover vast areas in a short time, but their high operational costs and limited access to certain terrains or airspace can be a drawback.

UAVs offer the flexibility of low-altitude data collection with reduced costs compared to aircraft. They excel in accessing challenging and remote terrains, making them ideal for localized mapping tasks. However, their limited payload capacity and flight time can restrict the area covered or limit the additional sensors to be used in combination with LiDAR.

Stationary pole or tripod-mounted LiDAR systems provide precise and repeatable data acquisition for monitoring specific crops. They are relatively cost-effective and easy to deploy, but their static nature limits their applications to localized areas. Stationary ground-based platforms are generally cost-effective and provide high accuracy. However, they might not be practical for mobile mapping or large-scale areas.

Mobile or stationary vehicles, such as tractors, ATVs, or cars, equipped with the appropriate mounting for LiDAR scanners, offer versatility for mobile mapping applications of agricultural or even forestry surveys. They can cover diverse terrains efficiently, but challenges arise from vehicle mobility, sensor stability, and occlusions in forested areas. On the other hand, CNC-based platforms provide precise control over LiDAR sensor movement, enabling custom scan patterns and high accuracy. However, this type of mechanism can be expensive or require extensive design work of hardware and software development, and also may require skilled or trained operators [9].

In conclusion, selecting the most suitable mounting system for LiDAR sensors involves carefully considering study requirements, budget constraints, and the specific challenges posed by each system. Aircraft and UAVs excel in large-scale and remote mapping tasks, whereas stationary options offer stability and precision for localized applications. Mobile vehicles and CNC platforms bridge the gap between mobility and precision but may come with higher costs and operational complexities. Understanding these differences and trade-offs is crucial for optimizing LiDAR data acquisition and achieving successful outcomes.

### 3.5. Additional Sensors and Instrumentation

Additional instruments were used in the different articles, complementing the typical facilities provided by LiDAR technology. Most of such instruments consist of imagery systems (e.g., rgb and hyperspectral cameras) employed to complement the original models obtained from LiDAR data acquisition. Next, we summarize some of the most relevant instruments along with a brief description, their different alternative models, and their usage:

- **RGB cameras**: Between the used devices, digital cameras are the most affordable instrument. Almost every smartphone today has a built-in RGB camera with a resolution that matches most of the requirements for the purpose of processing crop images or performing colorimetric analysis. Furthermore, recreational UAVs incorporate built-in cameras and are able to record video in high definition, making it available for later processing. Some of the models mentioned in the studied bibliography are Nikon D200 (digital camera) [18], Canon Digital EOS 5D (digital camera) [31], PointGrey FL3 (high-speed video camera) [32], Phantom 3 (drone) [33], DJI FC6310 (drone) [34], and Sony a7R III (digital camera) [35]. An example of application of this technology was presented in [36], where the authors propose a tassel detection scheme in corn plants using an airborne RGB camera (Sony Alpha 7RIII).
- **Thermal cameras**: This type of camera works with long wavelength infrared spectrum (14 μm) that allows for measuring the heat emitted by objects as radiation [37]. This type of camera was used in [32], particularly the Keii MC1-640 (IPI Infrared). Even though this last camera model is typically designed for UAV mounting, the authors in [32] used a custom stationary terrestrial scanning platform.
- **Hyper-spectral cameras**: This imagery system allows for measuring multiple vegetation indexes, as they measure hundreds of different wavelengths from the electromagnetic spectrum [37]. In [38], authors included the use of the Penta-Spek (developed by the Julius Kühn Institute) to acquire hyper-spectral data from the plots of barley. In the same line, the Compact Airborne Spectrographic Imager (CASI) is a hyper-spectral sensor intended for use with light aircraft. CASI was developed by Itres Research Ltd. (Calgary, AB, Canada) in 1988 and was designed for a variety of remote-sensing applications in forestry, agriculture, land-use planning, and aquatic monitoring [39,40,41].
  Another example of a hyper-spectral camera is the BaySpec OCI-UAV-1000 [32], which was mounted on a terrestrial stationary platform. This is a hyper-spectral camera for use on UAVs. Finally, in more recent research, we find the UAV-mounted hyper-spectral sensor (Headwall Nano-Hyperspec VNIR) mentioned by [35].
- **Multi-spectral cameras**: Multi-spectral cameras are cameras that can photograph the environment with a limited number of spectra in the visible and infrared spectrum [37]. In [34], a UAV-mounted Parrot Sequoia (MicaSense Inc., Seattle, WA, USA) was used to estimate Above-Ground Biomass (AGB).
- **Spectrometers**: These are instruments that can sense the amount of light reflecting from objects. They measure light in the visible (400–700 nm) and infrared spectra (700–2500 nm). Spectral sensors are used widely in agriculture because it has been found that these measurements are related to a plant’s physiology and development. In [42], eight spectral reflectance sensors (Meter Group Inc. Pullman, WA, USA) were used to measure reflected radiation in wheat plots.
- **Reflectance panels**: In order to calibrate LiDAR scanners to correctly operate given the environmental factors (temperature, humidity, etc.), high reflectance panels are used as special targets (e.g., the Spectralon (Labsphere, Inc., North Sutton, NH, USA) [43]).
- **Optical sensors**: These sensors detect electromagnetic radiation that falls within the visible spectrum, i.e., between infrared and ultraviolet wavelengths. In [20], an active optical sensor was used, namely the Raptor ACS 225LR (Holland Scientific, Holland Scientific Inc., Lincoln, NE, USA). This sensor was mounted in a passenger car along with the LiDAR scanner in order to measure biomass in fescue pastures. Another example of the usage of optical sensors is [42], where photodiodes paired with interference filters are used to measure PRI (Photochemical Reflectance Index) for the phenotyping of wheat plants.
- **Canopy analyzers**: The instrumentation that falls into this category combines different sensors (such as spectrometers or optical sensors) in order to measure certain properties of leaves and plant canopies (e.g., LAI, NDVI, NDRE, among many others). In [44], a leaf area index meter (LAI-2000, LI-COR Inc., Lincoln, Nebraska) and leaf area meter (LI3000, LI-COR Inc.) were used to measure leaf area in maize plants. A similar instrument (LAI-2200 Li-COR, Inc.) was used in [40,45] for the same purpose. In the same line, an NDVI sensor, the GreenSeeker^®^ (Trimble, USA), was used in [46] to estimate ground cover. Although this instrument is an active spectral sensor, it is specifically designed to measure NDVI in vegetation. Another crop-specific instrument is the ceptometer, which is a type of analyzer that measures the photosynthetically-active radiation that is reflected by plant leaves. A ceptometer (Decagon Devices, Inc., Pullman, WA) was used in [16]. Finally, in [23], a crop sensor, the RapidSCAN CS-45, was used. This device measures NDVI, NDRE, and reflectance indexes, among others, and is equipped with GPS.
- **Satellite imagery**: As part of additional instruments, we include the satellite images, as they are acquired through additional systems apart from the LiDAR scanners. In [44], GF-1 (Gaofen-1) data were used to estimate biophysical parameters in maize fields.

### 3.6. Software

Depending on which point of the workflow was applied, we can classify the used software in three categories: *data acquisition*, *data processing*, and *data visualization*. First, LiDAR data are acquired through the instrument itself, so specific data acquisition software is required. The data processing stage is the more complex and for some cases, the development of custom software is required in order to implement specific algorithms. Finally, a data visualization stage is usually included to assess the quality of the point clouds or 3D-generated models. In this case, the development of custom software is more complex, but usually there is the availability of specific programs and formats for this task. It must be noted that some manufacturers provide the software necessary for each stage, and also custom software development may be involved.

Below, we describe the software that was mentioned in more than two articles, among the analyzed papers, including a brief description of each one. The complete list of software used in each article can be found in Table A1.

- **General purpose software:** In this category we can enumerate software products that are currently being used in many fields, particularly when dealing with sensors and similar equipment. Thus, Labview (National Instruments) was used by authors of six different articles to implement the data acquisition stage. For data processing and data visualization, Matlab (Mathworks), R (R Development Core Team), and Excel (Microsoft) was used in nine, seven, and four articles, respectively. In [31,47], AutoCAD (Autodesk) was used as a data and model visualization tool. Most recent articles have incorporated machine learning tools, as the case of [48], where H2O-AutoML was used to classify data.
- **Scanner companion software:** Riegl RiSCAN PRO is the companion software for Riegl TLS Systems and was used in several papers. According to the manufacturer, “the entire data acquired during a measurement campaign can be organized and stored in the software’s project structure. These data include scans, fine scans, digital images, GPS data, coordinates of control points and tie points, and all transformation matrices necessary to transform the data of multiple scans into a common well-defined coordinate system”. Riegl RiPROCESS was also developed by the same manufacturer, and this software is designed for managing, processing, analyzing, and visualizing kinematic data acquired with airborne laser scanning systems based on Riegl Laser Scanners. It was used in [34,49], where the Riegl VUX-1UAV airborne laser scanner was employed. Another TLS companion software is Faro Scene (which was mentioned in the six papers that used Faro scanners, except in [32]), where a custom software called Crop3D was developed by the authors to implement specific algorithms.
- **Point cloud data processing and visualization:** Lastly, we mention specific software applied to 3D point cloud data analysis. In four articles, the authors use TerraScan (Terrasolid), which offers project structuring tools and automatic filtering algorithms. In the cases of ArcGIS, a mapping and analysis solution, and ENVI, a specialized software in geospatial image processing and analysis, both were developed by ESRI and were used in three and two articles, respectively. LiDAR360 (Geosystems Ingeniería), which was used in [34,49,50,51], is a post-processing software that includes a set of tools to visualize, manipulate, and generate geospatial-based products from point cloud data. OPALS (Orientation and Processing of Airborne Laser Scanning) was developed by the Technische Universität Wien, and according to its authors, it provides a processing chain for airborne laser scanning data (waveform decomposition, quality control, georeferencing, structure line extraction, point cloud classification, and DTM generation) and has several fields of application like forestry, hydrography, city modeling, and power lines. It was mentioned in [52,53]. CloudCompare, which is an open-source project, is applied to 3D point cloud and mesh processing. It was used in [23,42]. Finally, Photoscan Professional (Agisoft) was used in [34,54]. It is a standalone software product for performing photogrammetric processing of digital images and the generation of 3D spatial data. It is also worth mentioning that recent articles propose different algorithms to improve the accuracy of LiDAR sensors, for example in autonomous data acquisition vehicles, by combining data from multiple sensors such as inertial (R-INS) and navigation (GNSS) sensors [55].

## 4. Challenges and Recommendations

**Figure 3:**
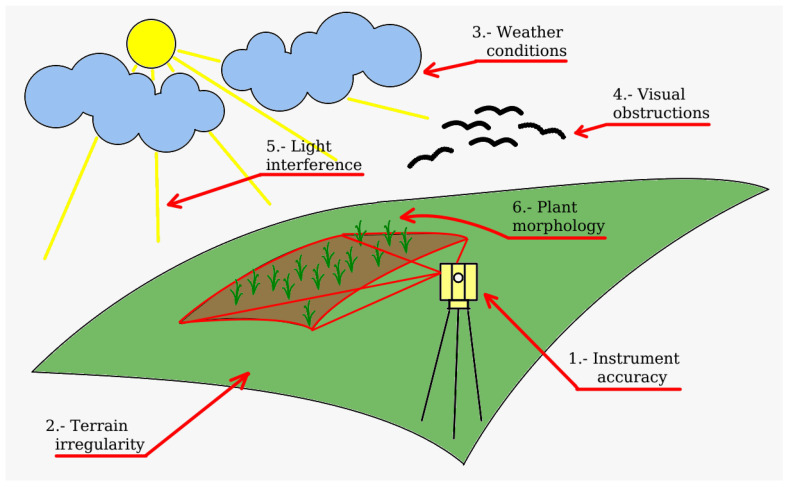
Identification of main challenges when using laser scanning data acquisition systems.

The use of LiDAR technology to analyze herbs, ground crops, and cover crops imposes certain challenges that should be taken into account. Some of these challenges are not readily apparent and most of them are discovered when performing the experiments for the first time. In this section, we analyze the most frequent difficulties and how to overcome them as proposed in the different research articles considered in this review. Figure 3 shows a visual summary of the main items detailed below.

### 4.1. Instrument Accuracy

When speaking about distance measurement using LiDAR devices, the footprint size of the laser beam is one of the factors that determine the accuracy of the sensor. As the beam is projected from the light emitter with a conical shape, the footprint size is given by the intersection of this cone with the reflective surface. It must be noted that the bigger the footprint size, the larger the detectable target should be; this introduces possible discrepancies that may affect the performance of the instrument for some applications.

Another factor that affects accuracy is the reflectance index of the surfaces being measured. The higher the value of the reflectance index of a given target, the lower the error will be when measuring the distance to that target. Related to the surface being measured, its inclination angle with respect to the laser beam trajectory also contributes to the measuring error. Given that the inclination angle of the surfaces corresponding to vegetation is in general randomly distributed, in order to achieve a higher accuracy it is recommended to have previous knowledge of this distribution and orient the instrument in a way the laser beam is mostly perpendicular to the vegetation structure [16,30].

### 4.2. Ground Level and Terrain Irregularity

Ground level and detection is one of the most mentioned issues and becomes particularly relevant for those applications where it is necessary to measure crop height. For instance, the accuracy when measuring the ground level is frequently mentioned in [11] as an important issue for the estimation of crop volume.

When the extension of the crop analyzed is small, the terrain model (or DTM, Digital Terrain Model) is usually assumed to be flat (i.e., a horizontal plane) [56]. For larger extensions of crop—as in the case of airborne laser scanning—hills, slopes, and valleys in the terrain should be taken into account, as such geographical accidents may affect the accuracy of the height measurement of the plant species involved.

In some cases, the ground level is measured manually [12,14,42,57]. This is mostly feasible in terrestrial laser scanning systems, because the distance between the laser scanner and the ground is measurable. However, terrain irregularity affects mobile platforms or robot-based terrestrial scanning systems because it introduces additional noise (given the movement or vibration of the scanner). This condition can be improved by hardware, using stabilizers such as gimbals (or alternatively, as proposed by most of the authors in the analyzed bibliography, by specific software based on denoising algorithms). In [19], for example, a terrestrial rover was used, and in order to eliminate the error caused by changes in the inclination angles of the scanner, a correction algorithm was developed that improved the measurement accuracy. In a similar way, in [13], a ground detection algorithm was introduced in order to reduce errors due to terrain irregularity. Reducing the size of the point cloud was also an alternative, as performed in [58], where the bottom part of the point cloud was deleted (with a threshold of 0.005 m).

A simple way to measure the terrain model is by performing a scan prior to the growth of the crops and assuming an invariant terrain model over time [16,18]. Another method is to assume that points in the point cloud data that fall within a certain range correspond to the ground, as proposed by [9,59,60]. Finally, the combination of the detection of ground returns and the data-point heights may be used to classify the points corresponding to the ground surface, as proposed, for example, in [54].

LiDAR systems that employ full-waveform return allow for detecting ground points from information that is present in the return signal itself. In [44,61], a specific commercial software (TerraScan) was used to classify point cloud data into ground points and non-ground points. In addition to the use of this software, a method of faint returns retrieval was also proposed in [45] to detect and obtain ground returns. With the same purpose, alternative software products were used, such as the Lidar360 software [34] and the Faro Scene software [62].

In [35], a commercial software (manufacturer not mentioned) was used to generate DTM and DSM, applying a ground filtering algorithm to separate bare earth points and aboveground points.

In [50], an “improved progressive triangulated irregular network densification filtering algorithm” was used to classify ground points and non-ground points, and a digital terrain model of 5 cm resolution was calculated from the LiDAR ground returns using the “ordinary kriging method”.

### 4.3. Weather Conditions

The weather dependency of the scanning process imposes another important drawback of the LiDAR technology in general, as meteorological conditions may prevent performing an evenly time-spaced sampling acquisition process. In particular, for some places with bad weather conditions, many days may go by without a measurement being made.

The wind is mentioned many times as an uncontrolled negative effect that introduces noise and increases the measurement error affecting the accuracy of acquired models and measured or estimated variables [22,32,35,58,59,63,64]. For this reason, several authors (such as [19,28,34,60,62]) stress the fact that outside experiments were performed on calm, sunny days. Windless conditions are also important for the case of UAV-based scanning, for flight stability reasons. In [64], it is stated that when flying at low altitudes, UAVs may produce downwash winds that move the plant canopies.

Humidity also affects the properties of air as the medium through which light travels. Some models of LiDAR sensors are prepared to withstand bad climatic conditions and have configurable parameters to correct possible measurement errors, but it is desirable to perform scans without fog or rain.

In [65], a fog filter was used, which is less sensitive in the near range (up to approximately 4 m). Not all LiDAR sensors seem to be affected by bad weather conditions. For example, according to [13], the FX6 Nippon LiDAR sensor is not influenced by sunlight or other weather conditions and is possible to operate in 24 h handling conditions like light, fog, and dust.

### 4.4. Visual Obstruction Problems: Birds, Insects, and Small Airborne Particles

When performing LiDAR data acquisition outdoors, especially in places where the presence of insects or birds prevails, the field of view of the instrument may be temporary and unnoticeably obstructed, and therefore, the acquired model may contain errors. The use of glass or plastic domes is not recommended by manufacturers as it may affect the speed of light and phase of the traveling light pulse. Some difficulties related to insects or dust were mentioned in [59]. In other cases, outliers can be produced by particles in the air, and are to be manually removed [53,60].

### 4.5. Sunlight and Light Interference

Another drawback that arises from making measurements outdoors is the light interference [66]. As can be observed from Table 3, the wavelength of some LiDAR instruments is near the visible spectrum (380–740 nm). Most commercial devices are prepared to work in sunlight conditions. However, in [9] a very low-cost LiDAR sensor and slow CNC mechanism were used, providing a closed structure to protect the laser sensor from sunlight and wind.

### 4.6. Plant Morphology

The accuracy of most LiDAR sensors depends on the shape of the object being scanned, its distance to the scanner sensor, the object’s reflective index, and its angle, as was previously mentioned in Section 4.1. However, none of the reviewed articles include species with well-differentiated features (for example, combining sunflower and wheat). It must be observed that testing different species with LiDAR technology requires different instrument configurations and algorithm settings.

In [16], plant morphology was analyzed in more detail, as different geometries were tested as ways of modeling maize leaves. In [47], it was mentioned that vegetation different from the one of interest, as weeds, were previously removed “to avoid interferences”.

In conclusion, LiDAR technology presents an immense potential for a wide range of applications, including remote sensing and environmental monitoring or assessment. However, a successful utilization of LiDAR requires a deep understanding of the various factors that can have an impact on data acquisition processes. From instrument-related considerations (such as footprint size, reflectance index, and inclination angles) to environmental factors (such as weather conditions, visual obstructions, and interference), each element plays a critical role in the quality of LiDAR data. Additionally, the terrain irregularity and ground level detection are essential for accurate measurements, especially in crop height estimation. Mitigating these challenges often involves a combination of hardware enhancements and advanced software algorithms. Despite these complexities, researchers and practitioners have made significant strides in overcoming obstacles, making LiDAR an increasingly valuable tool in diverse fields. Continued advancements in LiDAR technology, combined with robust methodologies for data processing and interpretation, promise a bright future for harnessing the full potential of LiDAR in addressing complex real-world challenges. By refining data acquisition strategies and continually innovating in this field, LiDAR will undoubtedly play a pivotal role in shaping our understanding of the environment and advancing numerous industries in the years to come.

## 5. Conclusions

In this work, a total of 53 research papers published between 2005 and 2022 was comprehensively reviewed in order to assess the state-of-the-art in ground crop analysis using LiDAR technology. Our analysis allowed us to identify different categories associated with LiDAR, providing a taxonomic view based on possible application areas, crop species under analysis, scanner technologies, and mounting platforms being used, additional sensors and instrumentation for expanding LiDAR capabilities, and specific software packages (such as data acquisition, data processing, etc.).

The proposed categories in this survey article have helped identify different salient aspects of LiDAR technology in the context of real-world applications. Thus, three particular application areas were identified (general purpose 3D reconstruction, parameter characterization of crops, and automatic control for agriculture machinery). We could also determine the proportion of different crop species under analysis, and the role of the two major LiDAR scanning technologies (ToF and PS), as discussed in Section 3. When analyzing the different mounting platforms, we provided a specific taxonomy for classifying those platforms according to their features (terrestrial, airborne, stationary, mobile, UAV, etc.), establishing as well their proportion of usage. From the analysis of additional sensors and instrumentation, we were able to identify a wide range of devices that can be used to complement LiDAR technologies in order to improve their performance. Similarly, we identified different specific software tools used with these technologies (such as general-purpose software, scanner companion software, and point cloud data processing and visualization).

The taxonomic view provided in this survey helped also to identify existing challenges when deploying LiDAR technology in real-world scenarios (such as instrument accuracy, terrain irregularity, weather conditions, visual obstruction problems, and plant morphology), as discussed in Section 4. In our opinion, the trade-off between the economic cost of LiDAR scanners and the agronomically relevant information that can be acquired with such instruments seems to limit the wide adoption of this technology. This might be one of the main reasons why these technologies are being actively researched for academic purposes but not massively used yet in agriculture landscaping. Given this observation, and assuming that the cost of the LiDAR technology will remain constant, the only alternative for these techniques to be applied in ground crop production is to increase the added value of the information that can be acquired from scanning ground crop fields. This is the aim of most of the reviewed articles and seems to be the current trend in this emerging area.

Finally, it is interesting to note that none of the articles from the reviewed bibliography tackle the problems associated with working with multiple species with the same setup and configuration, as all instrument and algorithm calibrations were fine-tuned to improve the performance of proposed methodologies in very specific situations. An interesting question to be considered for future research is the possibility of defining a general-purpose LiDAR-based framework that could be suitably parametrized for working with multiple species simultaneously.

## Figures and Tables

**Table 1:** Categories used to classify the different articles.

Category	Description
*Application areas*	This sorting criterion allowed us to identify three major sub-categories related to LiDAR technologies, namely 3D reconstruction, parameter-based characterization of crops, and automatic control for agricultural machinery.
*Analyzed crop species*	This criterion refers to the different species of ground crops under study when LiDAR technologies were applied.
*LiDAR scanner technology*	Scanners may vary in brand, model, and specifications. We surveyed the most relevant scanner technologies for LiDAR and their salient features.
*Mounting platform*	This category refers to different features that characterize mounting platforms for LiDAR scanners (e.g., terrestrial or airborne, stationary or mobile, etc.) as well as different platform types.
*Additional sensors and instrumentation*	In many cases, LiDAR scanners are enhanced through the addition of specific sensors and instruments (such as thermal cameras, spectrometers, etc.). In this category, we identified such sensors and instruments and discuss briefly their applicability.
*Software*	LiDAR technology relies on different software packages used in data acquisition, data processing, or data visualization. In this category, we discuss such software packages and tools.

**Table 2:** Agriculture-/canopy-/vegetation-related (left) and technical (right) acronyms and their corresponding meanings.

Abbreviation	Meaning	Abbreviation	Meaning
AGB	Aboveground Biomass	ALS	Airborne Laser Scanner
BGB	Belowground Biomass	ATLS	Autonomous Terrestrial Laser Scanner
CC	Canopy Cover	AOS	Active Optical Sensor
CHM	Canopy/Crop Height Model	CFI	Comparative Fit Index
CSM	Crop Surface Model	DART	Discrete Anisotropic Radiative Transfer
DBH	Diameter Breast Height	GFI	Goodness of Fit Index
DEM	Digital Elevation Model	GNSS	Global Navigation Satellite System
DSM	Digital Surface Model	HTPP	High-Throughput Phenotyping Platform
DTM	Digital Terrain Model	ICP	Iterative Closest Point
FVC	Fractional Vegetation Cover	LiDAR	Light Detection and Ranging
GAI	Green Area Index	LPI	Laser Penetration Metric
GC	Ground Cover	MEMS	Micro-Electro-Mechanical Systems
GSD	Ground Sample Distance	NIR	Near InfraRed
LAI	Leaf Area Index	OPALS	Orientation and Processing of Airborne Laser Scanning Data
LAD	Leaf Area Density	PDGPS	Phase Differential Geographic Positioning System
LAYM	Look Ahead Yield Monitor	PS	Phase Shift
LIA	Leaf Inclination Angle	RANSAC	Random Sample Consensus
LWA	Leaf Wall Area	R-INS	Reduced Inertial Navigation System
NDRE	Normalized Difference Red Edge	SEM	Structural Equation Modeling
NDVI	Normalized Difference Vegetation Index	SfM	Structure from Motion
NNI	Nitrogen Nutrition Index	SRI	Spectral Reflectance Indices
PAD	Plant Area Density	SRS	Spectral Reflectance Sensor
PRI	Photochemical Reflectance Index	TLS	Terrestrial Laser Scanner
ROI	Region of Interest	ToF	Time of Flight
SSWM	Site-Specific Weed Management	UTM	Universal Transverse Mercator
TAI	Tree Area Index	VNIR	Visible and Near InfraRed
TRV	Tree Row Volume		

**Table 3:** Top ten used devices and their specifications.

Brand/Model	Articles	Method	Wavelength (nm)	Pulse Rate (khz)	Range (m)	Accuracy (mm)	Power (w)	Weight (kg)
Sick LMS400	5	PS	650	0.5	3	3	25	2.3
Leica ALS70	5	ToF	1064	500	4412	380	972.4	107
Riegl VZ-400	4	PS	1545	300	600	5	80	9.6
Sick LMS111	4	ToF	905	0.05	20	30	8	1.1
Pulstec TDS-130L	3	N/D	N/D	N/D	9.5	N/D	N/D	N/D
Sick LMS291	3	ToF	905	0.075	80	10	20	4.5
Faro Focus X330	3	PS	1550	1350	330	2	40	5.2
Riegl VZ-1000	2	ToF	1550	122	1400	8	75	9.8
Faro Focus X120	2	PS	905	976	120	2	40	5
Leica Scanstation 2	2	ToF	400–700	50	300	6	80	18.8

## Data Availability

Data sharing not applicable.

## Appendix A

**Table A1:** Bibliographic references under analysis and their features.

Ref.-Year	Crop Species	Device Model	Mount/Mobility/Platform	Application	Software Involved
[16]-2005	maize, sunflower	OPTECH LiDAR	Airborne/Mobile/Aircraft	Param. characteriz.	-
[17]-2008	wheat, oat, barley	Faro scanner	Terrestrial/Mobile/Platform	Param. characteriz.	Faro Scene
[18]-2009	sugar beet	Riegl LMS-Z420i	Terrestrial/Stationary/Tripod	Param. characteriz.	Riegl RiSCAN PRO + ArcGIS
[27]-2009	wheat	Pulstec TDS-130L	Terrestrial/Stationary/CNC	Param. characteriz.	-
[28]-2009	wheat	Pulstec TDS-130L	Terrestrial/Stationary/CNC	Param. characteriz.	-
[11]-2009	wheat	SICK LMS200 + SICK LMS400	Terrestrial/Mobile/Tractor	Automatic control	Labview
[12]-2010	maize	IBEO ALASCA XT	Terrestrial/Mobile/Tractor	Automatic control	Custom software + Excel
[13]-2011	maize	Nippon-Signal FX6	Terrestrial/Mobile/Rover	Automatic control	Custom software + Gazebo
[29]-2012	rice	Pulstec TDS-130L	Terrestrial/Stationary/Tripod	Param. characteriz.	-
[19]-2012	miscanthus giganteus	SICK LMS291	Terrestrial/Stationary+Mobile/Tractor	Param. characteriz.	-
[14]-2013	maize	SICK LMS291	Terrestrial/Mobile/Rover	General purpose	Labview + Matlab
[31]-2013	maize	SICK LMS111	Terrestrial/Stationary/CNC	General purpose	Matlab + AutoCAD
[47]-2013	maize	Hokuyo URG-04LX	Terrestrial/Mobile/ATV	Param. characteriz.	Labview + AutoCAD
[67]-2014	maize	Hokuyo URG-04LX-UG01	Airborne/Mobile/UAV	Param. characteriz.	Robotic Operating System (ROS)
[52]-2014	rye, wheat	Riegl VZ-400	Terrestrial/Stationary/Platform	General purpose	Riegl RiSCAN PRO + OPALS
[59]-2014	rice	Riegl VZ-1000	Terrestrial/Stationary/Tripod	Param. characteriz.	Riegl RiSCAN PRO + ArcGIS + Excel + OriginPro
[63]-2014	maize	Riegl VZ-400	Terrestrial/Stationary/Tripod	General purpose	Riegl RiSCAN PRO
[43]-2014	wheat	Leica ScanStation 2	Terrestrial/Stationary/Tripod	Param. characteriz.	R + Cyclone + Interactive Data Language
[57]-2015	maize	SICK LMS291	Terrestrial/Mobile/Rover	General purpose	Labview + Matlab
[44]-2015	maize	Leica ALS70	Airborne/Mobile/Aircraft	Param. characteriz.	ENVI + TerraScan
[61]-2015	maize	Leica ALS70	Airborne/Mobile/Aircraft	Param. characteriz.	TerraScan + AMOS
[38]-2015	barley, other weeds	Riegl VZ-400	Terrestrial/Stationary/Platform	General purpose	Riegl RiSCAN PRO
[65]-2015	maize	SICK LMS111	Terrestrial/Mobile/Rover	General purpose	Matlab
[68]-2016	wheat	optoNCDT ILR 1191, Micro-Epsilon	Terrestrial/Stationary/Platform	Param. characteriz.	Interactive Data Language + R
[45]-2016	maize	Leica ALS70	Airborne/Mobile/Aircraft	Param. characteriz.	TerraScan + FV-2000 (LI-COR 2010)
[20]-2016	fescue	SICK LMS151	Terrestrial/Mobile/Car	Param. characteriz.	Excel
[64]-2016	maize, soybean, wheat	Faro Focus S120	Terrestrial/Stationary/Tripod	Param. characteriz.	Faro Scene + Matlab
[53]-2016	maize	Riegl VZ-400	Terrestrial/Stationary/Platform	Param. characteriz.	OPALS + Riegl RiSCAN PRO
[40]-2017	maize	Leica ALS70	Airborne/Mobile/Aircraft	Param. characteriz.	TerraScan
[32]-2017	rice, sorghum, maize	Faro Focus X120	Terrestrial/Stationary/CNC	Param. characteriz.	Custom software
[54]-2017	wheat	SICK LMS400	Terrestrial/Mobile/Platform	Param. characteriz.	Matlab + Agisoft Photoscan Professional
[21]-2017	cotton	SICK LMS511	Terrestrial/Mobile/Tractor	Param. characteriz.	Labview + Matlab
[69]-2017	wheat	SICK LMS400	Terrestrial/Mobile/Rover	Param. characteriz.	-
[56]-2017	maize, wheat	Leica ALS50-II	Airborne/Mobile/Aircraft	General purpose	DART RTM + Blender
[33]-2018	maize, sorghum	Faro Focus X330 + Leica ScanStation 2	Terrestrial/Stationary/Tractor	Param. characteriz.	Leica + Faro Scene + Pix4Dcapture + Pix4Dmapper + Trimble’s Pathfinder Office + FUSION
[46] - 2018	wheat	SICK LMS400	Terrestrial/Mobile/Platform	Param. characteriz.	Custom software + PointCloud library
[60]-2019	wheat	Riegl VZ-1000	Terrestrial/Stationary/Tripod	Param. characteriz.	Riegl RiSCAN PRO + ArcGIS
[62]-2019	sorghum	Faro Focus X330	Terrestrial/Mobile/Tractor	General purpose	Faro Scene
[50]-2019	maize	Faro Focus X120	Terrestrial/Stationary/Tripod	Param. characteriz.	Faro Scene + LiDAR360 + WinFOLIA + Statistical Product and Service Solutions
[70]-2019	wheat	SICK LMS400	Terrestrial/Mobile/Tractor	Param. characteriz.	R + MiniGIS
[34]-2019	maize	RIEGL VUX-1UAV	Airborne/Mobile/UAV	Param. characteriz.	Agisoft PhotoScan Professional + ArcMap + ENVI + RiPROCESS + LiDAR360
[41]-2019	maize	Leica ALS70	Airborne/Mobile/Aircraft	Param. characteriz.	TerraScan + R
[22]-2019	wheat, American mint	RPLIDAR A2	Airborne/Mobile/Aircraft	General purpose	Matlab
[58]-2019	maize	Velodyne HDL64-S3	Terrestrial/Mobile/Rover	Param. characteriz.	Robotic Operating System (ROS) + Velodyne data acquisition
[71]-2020	wheat, barley	SICK LMS111	Terrestrial/Mobile/Tractor	Param. characteriz.	-
[9]-2020	wheat, barley, rye	VL53L0X	Terrestrial/Stationary/CNC	General purpose	Custom software + Matlab + Excel + InfoStat
[42]-2020	wheat	SICK LMS111	Terrestrial/Mobile/Platform	Param. characteriz.	Labview + CloudCompare + R
[49]-2020	maize	Riegl VUX-1UAV	Airborne/Mobile/UAV	Param. characteriz.	POSPac 7.2 (Applanix) + RiPROCESS + LiDAR360
[72]-2021	maize, wheat, soybean	Leica ADS100	Airborne/Mobile/Aircraft	Param. characteriz.	R
[35]-2021	maize, sorghum	Velodyne VLP-16 Puck Lite	Airborne/Mobile/UAV	General purpose	-
[23]-2021	beans, peas, barley	Faro Focus X330	Terrestrial/Stationary/Tripod	Param. characteriz.	Faro Scene + CloudCompare + R
[51]-2022	pasture	Faro Focus 3D S70 + Riegl VUX-1UAV	Terrestrial+Airborne/Stationary+Mobile/Tripod+UAV	Param. characteriz.	LiDAR360
[48]-2022	maize	Velodyne HDL32	Airborne/Mobile/UAV	Param. characteriz.	H2O-AutoML
